# The hemodynamic stability of remimazolam compared with propofol in patients undergoing endoscopic submucosal dissection: A randomized trial

**DOI:** 10.3389/fmed.2022.938940

**Published:** 2022-08-08

**Authors:** Yuwei Qiu, Wei Gu, Mingye Zhao, Yunyun Zhang, Jingxiang Wu

**Affiliations:** ^1^Department of Anesthesiology, Shanghai Chest Hospital, Shanghai Jiao Tong University, Shanghai, China; ^2^Outcomes Research Consortium, Cleveland, OH, United States

**Keywords:** gastrointestinal endoscopy, anesthesia, hypotension, enhanced recovery after surgery, hemodynamics

## Abstract

**Objective:**

Hypotension is common in propofol anesthesia. Whether remimazolam could reduce intraoperative hypotension remains unknown. We therefore tested the primary hypothesis that remimazolam reduces the incidence of intraoperative hypotension compared with propofol in adult patients undergoing endoscopic submucosal dissection (ESD) surgery.

**Materials and methods:**

We conducted a prospective trial to compare patients who received either remimazolam or propofol bolus induction and thereafter intravenous infusion. The hemodynamic parameters were measured using CNAP^®^ Monitor 500 system. Our primary analysis was to compare the incidence of hypotension defined as systolic blood pressure below 90 mmHg between remimazolam and propofol during the whole anesthesia period.

**Results:**

The incidence of hypotension decreased by 50%, from 67.9% in propofol group to 32.1% in remimazolam group (*p* < 0.01). Patients received less amount of intraoperative phenylephrine in the remimazolam group than the propofol group (0 [0–40] μg vs. 80 [0–200] μg, *p* < 0.01). Time-weighted average and cumulative time of hypotension was lower in remimazolam group compared with propofol group (*p* < 0.05). Cardiac output continuously measured by CNAP was preserved much better in remimazolam group compared with propofol group (*p* = 0.01), while systemic vascular resistance did not differ between the groups. The median time from discontinuation until full alertness was 4 [3–11.8] min in the remimazolam group compared with 15 [12.0–19.8] min in the propofol group (*p* < 0.01).

**Conclusion:**

Remimazolam has better hemodynamic stability than propofol in adult patients undergoing ESD surgery. The benefits of remimazolam on hemodynamic stability and hypotension prevention may be partly contributed to its better preservation of cardiac output.

**Clinical Trial Registration:**

[http://www.chictr.org.cn/com/25/showproj.aspx?proj=61104], identifier [ChiCTR2000037975].

## Highlights

- Remimazolam has better hemodynamic stability than propofol in adult patients undergoing ESD surgery.
- The benefits of remimazolam on hypotension prevention may be partly contributed to its better preservation of cardiac output.
- Remimazolam promotes faster recovery after surgery compared with propofol when antagonized with flumazenil.

## Introduction

Owing to advances in endoscopic techniques and favorable outcomes, endoscopic submucosal dissection (ESD) has become an established treatment for early esophageal cancer or mucosal disease, especially in patients without lymph node metastasis (1–3). Esophageal ESD is a relatively complex procedure, requiring precise maneuvers. Previous studies recommended that ESD should be performed under general anesthesia, with the aim to minimize patient movement, improve patients’ satisfaction, and reduce the occurrence of perforation or aspiration pneumonia. Therefore, general anesthesia is currently considered to be an optimal method for most ESD surgery (4).

Propofol, which has excellent sedative properties and a short terminal half-life, is commonly used in ESD surgery (5, 6). Nevertheless, propofol has some unfavorable adverse effects, including pain noted on intravenous injection and dose-related cardiovascular depression, especially when given in conjunction with opioids. Hypotension is the most frequent adverse events related to propofol use. It has been reported that the incidence of hypotension caused by propofol is as high as 31–50% in ESD surgery (7, 8). These side effects have led to the evaluation of new sedatives.

Remimazolam, an ultra-short-acting benzodiazepine hypnotic, has been used in the anesthesia of ESD (9). Remimazolam has a short half-life, which results in the quick onset and recovery. Most importantly, remimazolam has little depressive effect on cardiovascular system and can reduce the incidence of hypotension (10–12). At a dose of 1 mg/kg/h, remimazolam will provide anesthesia for operative surgery without major adverse effects. However, there is a scarcity of data to investigate the impact of remimazolam on occurrence of intraoperative hypotension compared with propofol.

In this study, we aimed to investigate the benefits of remimazolam on preventing hypotension compared with propofol during ESD surgery. Specifically, we tested the hypothesis that remimazolam reduces the incidence of hypotension compared with propofol using a continuous non-invasive arterial pressure monitor, CNAP^®^ Monitor 500 system (CNSystems Medizintechnik AG, Graz, Austria).

## Materials and methods

### Ethics and registration

This study was a prospective, randomized, parallel trial comparing remimazolam (HengRui Medicine Co., Ltd., Lianyungang, China) to propofol (Fresenius-Kabi AG, Bad Homburg, Germany) in ESD. Ethical approval for this study [IRB No. KS(Y)20230] was provided by the Institutional Review Board (IRB) of Shanghai Chest Hospital, Shanghai Jiao Tong University, Shanghai, China (Chairperson Ning Zheng) on 14 August 2020. Written informed consent was obtained from each patient before enrollment. The trial was registered before patients’ enrollment at http://www.chictr.org.cn/com/25/showproj.aspx?proj=61104 (ChiCTR2000037975, principal investigator: Jingxiang Wu, date of registration: 08 September 2020). Recruitment was extended from December 2020 to July 2021.

### Inclusion and exclusion criteria of patients

The inclusion criteria were as follows: (1) male and female subjects, aged 18–80 years; (2) ASA I–III; and (3) body mass index (BMI) between 18 and 30 kg/m^2^. Patients were excluded if they had (1) uncontrolled hypertension or hypotension, or clinically important coronary atherosclerotic heart disease or heart failure; (2) severe respiratory disease; (3) severe sinus bradycardia, or heart block, or frequent ventricular arrhythmia, or atrial fibrillation; (4) clinically important coagulopathy; (5) end-stage hepatic dysfunction or renal disease requiring dialysis; (6) emergent surgeries; (7) peripheral artery disease with upper extremities dysfunction; and (8) other occasions when the investigators determined inappropriate, including patients unsuitable to rapid extubate when ESD was used for superficial pharyngeal carcinoma.

### Randomization and masking

All eligible patients were randomized into one of the two groups, namely, remimazolam group and propofol group in a ratio of 1:1 by a computer-generated coding system. An opaque, sealed envelope was opened by a masked investigator to determine the group assignment after the patient had provided written informed consent. Outcome assessors and endoscopists were masked to the group assignment.

### Protocol

Patients were randomized to receive an initial dose of either remimazolam (0.3 mg/kg) or propofol (2.0 mg/kg) for the induction of anesthesia. According to the “Chinese Experts’ Consensus on the Diagnosis and Treatment of Sedation and Anesthesia in Digestive Endoscopy” (13), patients received their assigned treatment as intravenous push by a syringe in 1-min period. When the patient was sufficiently sedated [Modified Observer’s Assessment of Alertness/Sedation (MOAA/S) = 0, Supplementary Table 1] (14), induction of anesthesia was accomplished. If sedation was deemed to be inadequate defined as MOAA/S > 0, supplemental doses were administered as intravenous push by a syringe in 1-min period (remimazolam 0.1–0.2 mg/kg or propofol 0.5 mg/kg) until MOAA/S = 0. Then, sufentanil 0.5 μg/kg and rocuronium 0.6 mg/kg were given to facilitate tracheal intubation. After tracheal intubation, the anesthesia machine was connected for mechanical ventilation and the volume control mode was used.

Remimazolam or propofol was intravenously infused according to bispectral index (BIS) between 40 and 60. Remimazolam was initially infused at 1 mg/kg/h, with the maximum infusion rate of 3 mg/kg/h. When BIS exceeded 60, a supplemental dose of 0.15 mg/kg of remimazolam was then intravenously added; when BIS was below 40, remimazolam was decreased at a rate of 0.2 mg/kg/h step by step. Propofol was initially infused at 5 mg/kg/h. When BIS exceeded 60, a supplemental dose of 0.5–1 mg/kg of propofol was then intravenously added over 30 s; or propofol was decreased at a rate of 1 mg/kg/h when BIS was below 40. The infusion of remimazolam or propofol stopped when the endoscopic probe was withdrawn.

All ESD procedures in this trial were accomplished by two experienced endoscopists who specialized in ESD at least 2 or 3 years. Typically, ESD was conducted in a sequential step, including marking the perimeter of the lesion with cautery, and then injecting a lifting agent into the submucosa around the lesion, thereafter cutting circumferentially around the lesion, dissecting the submucosa beneath the lesion, and finally managing intraprocedural bleeding that occurred during mucosal incision or submucosal dissection.

Immediately after discontinuation of remimazolam or propofol, flumazenil 0.5 mg was injected to reverse the sedatives, and muscle relaxants were routinely reversed with atropine/neostigmine. An anesthesiologist determined when to extubate and evaluate patients’ recovery. When patients had an Aldrete score > 9 and felt warm-alert-comfortable, then they were allowed to discharge to the ward.

### Measurements

Patient was positioned in left lateral decubitus for ESD surgery. We attached the CNAP^®^ Monitor 500 system’s finger cuff to the finger of left hand and started the measurement after calibration. CNAP^®^ Monitor 500 system (CNSystems Medizintechnik, Graz, Austria) was a continuously non-invasive arterial blood pressure monitoring system with finger cuff-derived method, validated and utilized in various clinical settings (15). Systolic blood pressure (SBP), diastolic blood pressure (DBP), mean arterial pressure (MAP), heart rate (HR), cardiac output (CO), and systemic vascular resistance (SVR) at specific time-points, including baseline, 1 and 3 min after tracheal intubation, start of operation, every 5 min during the operation, and the end of operation were obtained from CNAP. Electrocardiography, pulse oxygen saturation, radical arterial invasive blood pressure, and esophageal temperature were monitored using GE Carescape Monitor B850 (GE Healthcare, Chicago, IL, United States).

### Outcome assessment

The primary outcome was the incidence of hypotension during the whole anesthesia period. Hypotension was defined as non-invasive systolic blood pressure (SBP) < 90 mmHg lasting at least 1 min. When hypotension occurred, phenylephrine 40 μg or more was intravenously administrated until SBP returned to the normal range (90–140 mmHg). The amount of phenylephrine was recorded.

The secondary outcome included the total amount of phenylephrine, time-weighted average and cumulative time of SBP < 90 mmHg, and time-weighted average and cumulative time of MAP > 100 mmHg. Time-weighted average of SBP under a threshold of 90 mmHg was calculated as the area between 90 mmHg threshold and the curve of the SBP measurements was divided by total continuous reading time (16). Time-weighted average of MAP > 100 mmHg was calculated by the same method. The time of first episode of hypotension was recorded.

We also recorded CO and SVR at the before-mentioned time points, as CO*SVR = (MAP-CVP) × 80; therefore, the product of CO and SVR could reflect the formation of MAP to some extent.

Emergence time was defined as the time from discontinuation of remimazolam or propofol to modified observer’s assessment of alert/sedation (MOAA/S) = 5 measured repetitively three times (Supplementary Table 1) (17). Time to extubate was defined as the time from discontinuation of remimazolam or propofol to the removal of endotracheal tube. Recovery time was defined as the time from discontinuation of remimazolam or propofol to the modified Aldrete score returning to 9 (18).

Patients’ demographics, surgical variables, anesthetic variables, pre-operative and post-operative arterial blood gases and electrolytes, and post-operative length of stay were measured and recorded.

### Statistical analysis

Continuous variables with normal distribution were expressed as mean ± SD, while data showing a skewed distribution were expressed as median (interquartile range). Categorical data were presented as number or percentages.

For baseline analysis and primary outcome, quantitative data between the two groups were analyzed by the two-sample *t*-test or non-parametric test, and categorical data were analyzed by χ^2^ test, or Fisher’s exact test.

For secondary outcome analysis, we compared the time-weighted average of SBP < 90 mmHg, cumulative time of SBP < 90 mmHg, time-weighted average of MAP > 100 mmHg, and cumulative time between the two groups using two-sample *t*-test or non-parametric test. Mann-Whitney *U* test was used to assess the difference between the two groups for non-normal distribution parameters, and χ^2^ test or Fisher’s exact test for binary outcomes.

Two-way repeated-measures ANOVA was used to assess the difference of CO and SVR at the before-mentioned time-points between the two groups. For recovery time and complications, the between-group comparisons of continuous or categorical data were analyzed by two-sample *t*-test or, Mann-Whitney *U* test, or χ^2^ test. *p* < 0.05 was considered statistically significant.

Our minimal sample size was determined as follows. The primary outcome of this study was the difference in the incidence of intraoperative hypotension between remimazolam and propofol. A previous study reported that the incidence of hypotension caused by propofol was as high as 31–50% in ESD surgery (7, 8). However, research was lacking regarding the incidence of hypotension following remimazolam treatment in ESD surgery, and thus, we conducted a pilot study. The result of our pilot study showed that the incidence of hypotension was 67% (4/6) in the propofol group compared with 25% (2/8) in the remimazolam group. We predicted that remimazolam could reduce the incidence of intraoperative hypotension from 67 to 25% in ESD surgery. Based on the assumption, a sample size of 44 patients had 80% power to detect a one-tailed 5% level of significance by G*POWER3.1.9 (22 per group). We estimated lost-to-follow-up of 20% and therefore increased the sample size by 20% (to 28 subjects per group) to allow for dropouts.

Analyses were performed using SPSS 22.0 (IBM, Chicago, IL, United States) and Python (3.9.10), with statistical significance defined by a two-sided *p* value of <0.05.

## Results

**FIGURE 1:**
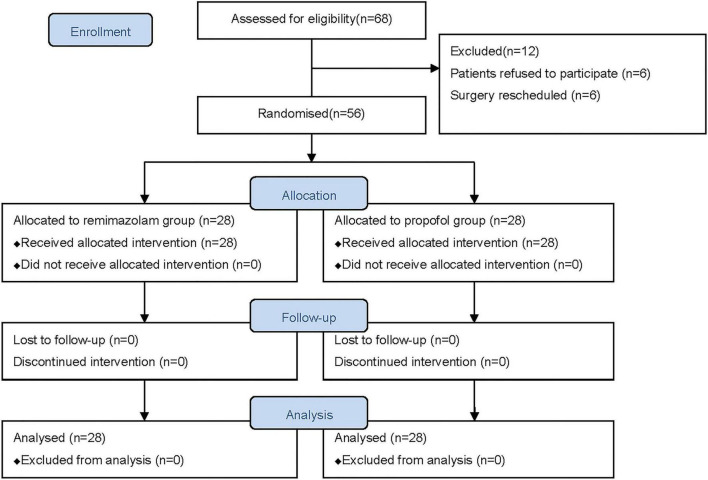
Flowchart of patients enrolled in the study.

We initially assessed 68 patients for eligibility, and 56 patients were finally enrolled and randomized to receive either remimazolam (*n* = 28) or propofol (*n* = 28). All patients completed follow-up and were included in the final analysis (Figure 1). The estimated intraoperative blood loss was less than 100 ml in all patients. The patients in the two groups were similar at baseline and surgical variables in terms of age, sex, body mass index, ASA physical status, history of hypertension and diabetes mellitus, pre-operative fasting time, duration of anesthesia, duration of surgery, IV fluid administration, pre-operative blood glucose and lactate, and esophageal temperature at the end of surgery (Table 1).

**TABLE 1:** Baseline characteristics of patients receiving remimazolam or propofol.

Variable	Remimazolam group (*n* = 28)	Propofol group (*n* = 28)	Standardized difference
**Demographic factors**			
Age (y)	62.8 ± 7.1	64.7 ± 8.9	0.24
Sex (male/female)	21/7	19/9	0.16
Body weight (kg)	65.1 ± 10.8	63.1 ± 8.5	0.20
Body mass index (kg/m^2^)	23.2 ± 3.5	22.3 ± 2.9	0.27
History of hypertension, *n* (%)	7 (25)	13 (46.4)	0.54
History of diabetes mellitus	3 (10.7)	3 (10.7)	0
History of alcohol (none/former/current)	10/13/5	15/9/4	0.23
History of smoke (none/former/current)	10/17/1	16/11/1	0.30
ASA physical status			0.18
I	3 (10.7)	1 (3.6)	
II	21 (75)	24 (85.7)	
III	4 (14.3)	3 (10.7)	
Pre-operative fasting time, hours	23 [20, 24]	21.5 [20, 24]	0.25
Pre-operative lactate level, mmol/L	1.14 ± 0.31	1.17 ± 0.35	0.09
Pre-operative glucose level, mmol/L	5.71 ± 0.67	5.55 ± 0.64	0.24
**Intraoperative factors**			
Total amount of sufentanil	35 [31.3, 40]	35 [30, 45]	0
Total amount of rocuronium	50 [40, 60]	50 [42.5, 65]	0
Duration of surgery (min)	53 [27.5, 81]	55 [35.5, 77.3]	0.09
Duration of anesthesia (min)	92.5 [66.3, 120.8]	87 [71.5, 114.8]	0.07
Colloid (mL)	200 [0, 500]	225 [50.0, 450]	0.10
Crystalloid (mL)	500 [500, 950]	500 [500, 700]	0.32
Estimated urine output, ml	Not applicable	Not applicable	
Esophageal temperature at the end of surgery (°C)	36.4 ± 0.38	36.4 ± 0.39	0.06

Data are presented as either mean ± SD, median [quartile 1, quartile 3], or number (%). The absolute standardized difference measures the mean difference between the remimazolam and propofol groups.

The primary and secondary outcomes are reported in Table 2. For the primary outcome, the incidence of hypotension was 32.1% in remimazolam group and 67.9% in propofol group. Remimazolam significantly decreased the incidence of intraoperative hypotension by 50% (*p* < 0.01). From anesthesia induction until the start of surgery, one of 28 (3.5%) patients developed the first episode of hypotension in the remimazolam group, compared with 10 of 28 patients (35%) in the propofol group (*p* = 0.002).

**TABLE 2:** Summary of blood pressure outcomes.

Outcomes	Remimazolam group (*n* = 28)	Propofol group (*n* = 28)	*P*-value
**Primary outcome**			
SBP < 90 mmHg, *n* (%)	9/28 (32.1)	19/28 (67.9)	0.008**
**Secondary outcomes**			
Total amount of phenylephrine, μg	0 [0, 40]	80 [0, 200]	0.001**
Time-weighted average of SBP < 90 mmHg	23.6 [0, 135.0]	99.1 [29.6, 276.5]	0.015*
Cumulative time of SBP < 90 mmHg, min	4.2 [0, 17.5]	13.1 [6.1, 29.1]	0.035*
Time-weighted average of MAP > 100 mmHg	0 [0, 2.0]	0.1 [0, 2.4]	0.801
Number of patients with any MAP readings > 100 mmHg	14/28 (50%)	15/28 (53.6%)	0.791
Cumulative time of MAP > 100 mmHg, min	0.9 [0, 14.7]	1.6 [0, 10.9]	0.876

Data are presented as either median [quartile 1, quartile 3], or number (%). Denotes statistically significant (*p < 0.05 or **p < 0.01) differences among the two groups. Mann-Whitney U test was used to assess the difference between the two groups for non-normal distribution parameters, and Chi-square or Fisher Exact tests for binary outcomes. SBP, systolic blood pressure; MAP, mean arterial pressure.

**FIGURE 2:**
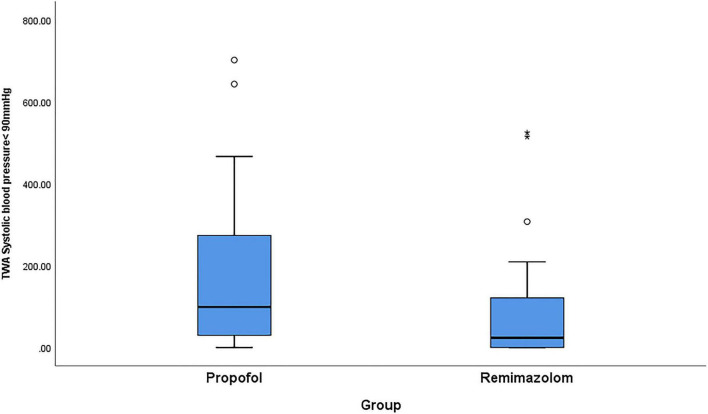
Comparison of propofol and remimazolam on time-weighted average of intraoperative hypotension. Boxplots are showed with the lines in the box represent median [Q1, Q3] of the observed TWA hypotension and the whiskers extended to the minimum at the bottom and the maximum on top. Abbreviation: TWA, time-weighted average. Intraoperative hypotension was defined as systolic blood pressure less than 90 mmHg. Q1 and Q3 represent 25th and 75th of the TWA hypotension.

**FIGURE 3:**
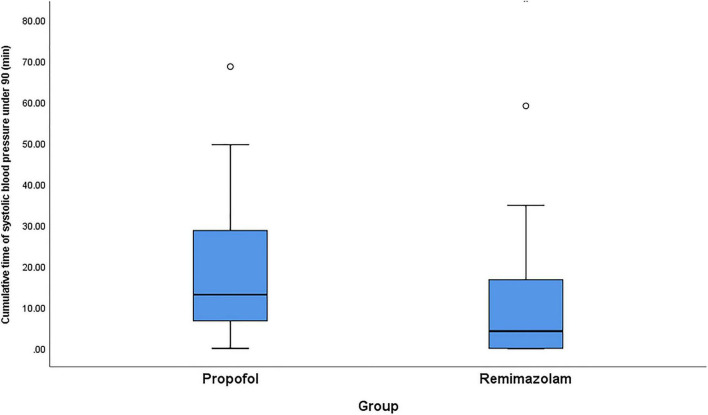
Comparison of propofol and remimazolam on cumulative time of intraoperative hypotension. Boxplots are shown with the lines in the box representing median [Q1, Q3] of the observed cumulative time and the whiskers extended to the minimum at the bottom and the maximum on top. Q1 and Q3 represent 25th and 75th of the cumulative time of intraoperative hypotension.

For the secondary outcomes, the total dosage of phenylephrine in remimazolam group was significantly less than that in propofol group, with the median of phenylephrine 0 [0–40] μg in the remimazolam group and 80 [0–200] μg in propofol group (*p* < 0.01). Time-weighted average of hypotension and the cumulative time of hypotension were much lower in the remimazolam group compared with the propofol group (Table 2 and Figures 2, 3). Time-weighted average and cumulative time of MAP > 100 mmHg was not significantly different between the two groups (*p* > 0.05) (Table 2).

**FIGURE 4:**
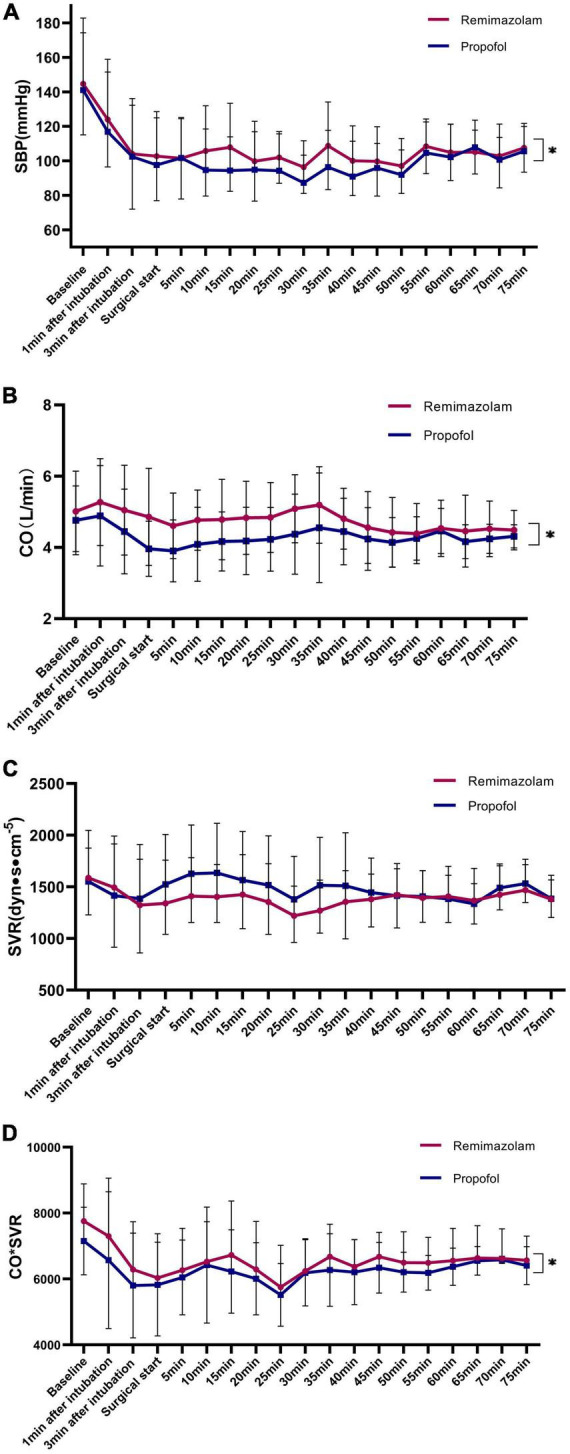
Trends of hemodynamic variables during surgery: **(A)** SBP; **(B)** CO; **(C)** SVR; **(D)** the product of CO and SVR. Data are presented as mean with error bars showing SD. Trends of SBP, CO, SVR, and the product of CO showing significant difference between groups (*p < 0.05). SBP, systolic blood pressure; CO, cardiac output; SVR, systolic vascular resistance; SD, standard deviation.

The trends of SBP, CO, SVR, and the product of CO and SVR at multiple measurement time points are shown in Figure 4. During the whole anesthesia period (around 75 min after surgery started), the trend of SBP was kept higher in remimazolam group compared with propofol group (*p* = 0.029, Figure 4A). CO preserved much better in remimazolam group compared with propofol group (*p* = 0.01, Figure 4B). In the remimazolam group, CO was kept stable at around 5 L, while in the propofol group, CO fluctuated continuously below 4.5 L, as shown in Figure 4B. SVR did not differ between the two groups (*p* = 0.126, Figure 4C). Although SVR showed a reduction in the remimazolam group, the difference did not reach statistical significance compared with that in the propofol group (shown in Figure 4C). The product of CO and SVR during the whole anesthesia period demonstrated a higher value in the remimazolam group compared with the propofol group (*p* = 0.001, shown in Figure 4D).

The incidence of injection site pain was 0% in the remimazolam group and 21.4% in the propofol group (*p* < 0.01) (Table 3). At the end of surgery, the level of blood glucose was 6.79 ± 1.38 mmol/L in the remimazolam group, compared with 6.01 ± 0.73 mmol/L in the propofol group, *p* = 0.030 (Table 3). Blood lactate levels were similar between the groups (Table 3).

**TABLE 3:** Clinical recovery variables and complications.

Variables	Remimazolam group (*n* = 28)	Propofol group (*n* = 28)	*P*-value
Emergence time, min	4 [3, 11.8]	15 [12.0, 19.8]	0.001**
Time to extubate, min	5 [3.0, 13.8]	15 [12.3, 20.0]	0.001**
Recovery time, min	5 [3.3, 12.5]	15 [13.3, 20.8]	0.000**
Post-operative hospital stays (d)	2 [1, 3]	2 [1, 3]	0.800
Injection site pain, *n* (%)	0 (0)	6 (21.4)	0.030*
Intraoperative atropine use, *n* (%)	0 (0)	3 (10.7)	0.236
Blood lactate at the end of surgery (mmol/L)	0.81 ± 0.21	0.89 ± 0.36	0.388
Blood glucose at the end of surgery (mmol/L)	6.79 ± 1.38	6.01 ± 0.73	0.030*

Data are presented as mean ± SD, median [quartile 1, quartile 3] or n (percentages). Denotes statistically significant (*p < 0.05 or **p < 0.01) differences among the two groups. Emergence time was defined as the time from discontinuation of remimazolam or propofol to MOAA/S = 5 measured repetitively three times; Recovery time was defined as the time from discontinuation of remimazolam or propofol to modified Aldrete score returned to 9.

The emergence time was 4 [3.0–11.8] min in the remimazolam group, much shorter than that in the propofol group, i.e., 15 [12.0–19.8] min (*p* < 0.01). Time to extubate was 5 [3.0–13.8] min in the remimazolam group and 15 [12.3–20.0] min in the propofol group (*p* < 0.01). Recovery time in the remimazolam group was 5 [3.3–12.5] min, which was shorter than 15 [13.3–20.8] min in the propofol group (*P* < 0.01).

One patient had post-operative agitation in the propofol group and none of the patients had post-operative agitation in the remimazolam group. Re-sedation occurred in 14.3% (4/28) of patients compared with 7.1% in both the groups (2/28), *p* = 0.669. Post-operative hospital stay in the two groups was not different (*p* = 0.800).

## Discussion

In this prospective, controlled study, we found that remimazolam had a clinically and statistically significant reduction of peri-anesthesia hypotension. Remimazolam decreased the hypotension by 50%, from 67.9% in the propofol group to 32.1% in adult patients undergoing ESD surgery, presenting a relatively stable cardiovascular profile. The total amount of intraoperative phenylephrine had a corresponding decrease. Our data showed that the benefits of remimazolam on hypotension prevention may be partly contributed to its better preservation of cardiac output during the whole period, without much reduction of systemic vascular resistance. Remimazolam also fastened recovery of patients after surgery compared with propofol.

Current anesthesia strategies recommend general anesthesia as a safer option for ESD (19). However, the fact which anesthetics or their combination is the better choice remains unclear in ESD. Although presenting acceptable sedative profile, propofol has a cardiovascular depression effect, resulting in a drop in blood pressure (20). Even a single shot of propofol during anesthesia induction can lead to the incidence of hypotension as high as 44% (21). Our study found that the incidence of peri-anesthesia hypotension caused by propofol was 67.9% during the 45 min to 1 h procedure. Previous trials mainly focused on the single-use of remimazolam on hypotension during induction (21–23). We continuously monitored the hypotension during the whole perioperative period and found that remimazolam decreased the hypotension by 50%, from 67.9% in propofol group to 32.1%. Remimazolam had the advantages of stable hemodynamics (24, 25), which may help explain why incidence of peri-anesthesia hypotension caused by remimazolam was much lower than propofol. Due to immediate treatment of hypotension, we had a corresponding increased amount of phenylephrine in remimazolam compared with propofol.

To find more information about hypotension, we also compared the TWA and cumulative time of SBP < 90 mmHg. We found that the severity and duration of hypotension was lower in remimazolam group compared with propofol group. Our data showed that remimazolam can reduce the incidence of post-induction hypotension and extend the time to first episode of hypotension. From post-induction until the start of surgery (around 14 min), one of 28 (3.5%) patients developed the first episode of hypotension in the remimazolam group, compared with 10 of 28 (35%) in the propofol group. We also investigated the effect of remimazolam on peri-anesthesia hypertension compared with propofol during the surgery and we found that it did not differ between the groups.

Our study was the first to compare the hemodynamic stability between remimazolam and propofol using the CNAP^®^ Monitor 500 system. Although previous studies found the stable profile of remimazolam in non-cardiac or cardiac surgery, the mechanism still remained uncertain. Interestingly, our data shed a light on the possible mechanism that how remimazolam benefited hypotension prevention and provided stable hemodynamics. Our result showed that remimazolam bolus injection and thereafter continuous infusion preserved better cardiac output than propofol. In the remimazolam group, we found that the cardiac output was kept stable above 5 L, while it fluctuated between 3 and 4.5 L in the propofol group. We also found that remimazolam had no significant reduction of systemic vascular resistance compared with propofol. While the product of cardiac output and systemic vascular resistance might greatly reflect the blood pressure, so, we made a plausible explanation that remimazolam prevented hypotension partly due to its better preservation of cardiac output as well as the product of cardiac output and systemic vascular resistance. As the median time of surgical duration was around 60 min, herein, we plotted the trend of cardiac output, systemic vascular resistance, and cardiac output*systemic vascular resistance in 75 min.

Remimazolam was metabolized rapidly into a non-active metabolite by non-specific esterase in the tissues (25, 26). A previous study showed that the full alertness was naturally regained 19 ± 7 min after stop of remimazolam infusion (27) without reversal. The hypnotic effect of remimazolam can be reversed by flumazenil, so remimazolam-treated patients had a quicker recovery from sedation after reversal by flumazenil. We found that the median time from discontinuation of remimazolam until full alertness was 5 min after reversal with flumazenil, which was similar to 3.5 min after one shot of remimazolam (22). No injection site pain was observed in the remimazolam group, compared with 21.4% that occurred in the propofol group, which was consistent with a previous study (28). Our data showed that patients given remimazolam infusion for 1 h had higher levels of blood glucose at the end of surgery compared with propofol. Our result was inconsistent with the finding of Liu (29), revealing that there was no significant difference in glucose values between propofol and remimazolam, following a one shot of 0.3 mg/kg (29). The change in blood glucose inadvertently found in our trial was perhaps an interesting finding. However, relevant literature about the effect of remimazolam on blood glucose was lacking, so we were not sure that this change of blood glucose was truly caused by the effect of remimazolam or just due to a small sample size. Large trials may be needed to address the question.

Our prospective, parallel control trial has some strengths to address our hypothesis. However, it still has some limitations. First, in this study, we used CNAP, the finger-application type, non-invasive hemodynamic monitors to detect hypotension and explore possible mechanism. The measurements of CNAP may be affected by exogenous vasoconstrictors. Second, we just included patients without severe cardiovascular diseases, so the conclusion cannot be generated to more elderly or fragile patients. Third, patients in both the groups had relatively long fasting time, which may exacerbate the occurrence of hypotension. Finally, our sample size was relatively small, so it may not exclude some potential confounders. The conclusion should be interpreted as conservative.

In summary, remimazolam has better hemodynamic stability and faster recovery than propofol in adult patients undergoing ESD surgery. Its efficacy in more generalized populations to prevent intraoperative hypotension remains to be further studied. The benefits of remimazolam on hypotension prevention may partly contribute to its better preservation of cardiac output during the whole period, without much reduction of systemic vascular resistance.

## Data availability statement

The raw data supporting the conclusions of this article will be made available by the authors, without undue reservation.

## Ethics statement

The studies involving human participants were reviewed and approved by the Institutional Review Board of Shanghai Chest Hospital, Shanghai Jiao Tong University School of Medicine, Shanghai, China [IRB No. KS(Y)20230] on August 14, 2020. The patients/participants provided their written informed consent to participate in this study.

## Author contributions

YQ contributed to the conceptualization, designing and writing the original draft, preparation, reviewing and editing, and statistical analysis. WG contributed to the collection and assembly of data, writing – original draft preparation, and reviewing. MZ contributed to the data analysis and collection and manuscript writing. YZ contributed to the data collection and manuscript writing. JW contributed to the conceptualization, methodology, administrative support, manuscript writing, reviewing, and editing. All authors have finally approved the manuscript.
