# Supplementary material for: The hemodynamic stability of remimazolam compared with propofol in patients undergoing endoscopic submucosal dissection: A randomized trial
Source: Front Med (Lausanne). 2022 Aug 8;9:938940. doi: 10.3389/fmed.2022.938940 (PMC9394743; doi:10.3389/fmed.2022.938940)
Supplement: Supplementary file 1 [file Table_1.docx]

| **Supplemental Table 1. Modified Observer’s Assessment of Alertness/Sedation** | |
| --- | --- |
| **Response** | **Score level** |
| Responds readily to name spoken in normal tone | 5 (alert) |
| Lethargic response to name spoken in normal tone | 4 |
| Responds only after name is called loudly or repeatedly | 3 |
| Responds only after mild prodding or shaking | 2 |
| Does not respond to mild prodding or shaking | 1 |
| Does not respond to noxious stimulation | 0 |
